# Systematic Analysis of Peripheral Immune Signatures and Diagnostic Model Construction in Patients With Uterine Fibroids

**DOI:** 10.1155/jimr/9688793

**Published:** 2026-01-17

**Authors:** Pei-Xian Li, Si-fei Yu, Yu-Bin Han, Xiao-hong Zhu, Kai-Rong Lin

**Affiliations:** ^1^ Institute of Translational Medicine, The First People’s Hospital of Foshan (Foshan Hospital Affiliated to Southern University of Science and Technology), Foshan, Guangdong, China; ^2^ Department of Gynecology, The First People’s Hospital of Foshan (Foshan Hospital Affiliated to Southern University of Science and Technology), Foshan, Guangdong, China

**Keywords:** diagnostic value, flow cytometry, immune cell subsets, peripheral immunity, uterine fibroids

## Abstract

**Objective:**

To systematically characterize the peripheral immune signature of uterine fibroids (UFs) and to develop a diagnostic model for differentiating UF patients from healthy individuals, thereby providing new insights into UF immunopathogenesis.

**Methods:**

We performed multiparametric flow cytometry analysis on peripheral blood samples from 31 UF patients and 63 age‐matched healthy controls (HCs). A total of 70 immune parameters were evaluated, encompassing T cells, B cells, natural killer (NK) cells, *γδ* T cells, and their functional subsets.

**Results:**

Comprehensive immunophenotyping revealed a distinct peripheral immune profile in UF patients. Key findings included a significant dysregulation within helper T (Th) cell compartments, characterized by elevated frequencies of functional Th and Th17 cells, alongside reduced proportions of senescent Th, T follicular helper 1 (Tfh1), and peripheral Th (Tph) cells. Concurrently, a significant expansion of the B cell compartment was observed, marked by increased total B cells, naïve B cells, immature regulatory B cells (Breg), and transformed B cells. In contrast, the frequencies and functional subsets of cytotoxic T (Tc) cells, *γδ* T cells, and NK cells showed no significant alterations after false discovery rate (FDR) correction. A random forest (RF) model incorporating key immune markers effectively discriminated UF patients from HCs, identifying several markers as central features with both diagnostic and mechanistic relevance.

**Conclusion:**

This study presents the first systematic atlas of the peripheral immune landscape in UF, revealing a pattern of systemic immune dysregulation centered on Th and B cell pathways. These findings advance our understanding of the immunopathogenesis of UF and establish a foundation for future immune‐based diagnostic and therapeutic strategies.

## 1. Introduction

Uterine fibroids (UF), the most prevalent benign tumors affecting the female reproductive system, have become a focal point of extensive concern due to their distinctive epidemiological traits and the daunting clinical hurdles they pose in management. Epidemiological investigations have revealed that the prevalence of UF among women of reproductive age ranges from 40% to 89% [1], with a significant positive correlation with age—reaching ~70% in women over 50 years [2]. Clinically, around 15% to 30% of affected patients exhibit severe symptoms, including dysmenorrhea, abnormal uterine bleeding, infertility, and constipation [3]. UF constitutes the primary indication for hysterectomy, accounting for 30% to 50% of all hysterectomy procedures [2]; however, this treatment has irreversible damage to the reproductive function of women of childbearing age. Alternative therapeutic strategies include laparoscopic or open myomectomy, uterine artery embolization, and image‐guided (radiological or ultrasound) interventions [3]; yet, these approaches are associated with high postoperative recurrence rates. Despite the high prevalence of UF, its underlying etiology remains largely elusive, resulting in extremely limited therapeutic options.

Early research has pinpointed multiple risk factors linked to UF, including early menarche, lifestyle factors (such as dietary habits, caffeine intake, and alcohol consumption), obesity, metabolic disorders, genetic predispositions, and immune dysregulation [3, 4]. In recent years, there has been a growing focus on the role of chronic inflammation and host immune responses in the initiation, progression, and recurrence of UF [5, 6]. Histopathological examinations have unequivocally demonstrated a marked disparity in the immune‐inflammatory microenvironment between UF lesions and matched myometrial tissues obtained from the same patient. Specifically, within UF lesions, there was a notable infiltration of immune cells, such as natural killer (NK) cells, total macrophages, M2–polarized macrophages, and conventional dendritic cells, along with a clear presence of inflammatory mediators like IFNA2, IL‐1*α*, and PDGF‐AA [7]. Recent single‐cell sequencing analyses have further confirmed that the UF microenvironment exhibits a distinct immune infiltrative profile, enriched with activated T cells, NK cells, and other immune subsets [8]. The alteration in the immune microenvironment not only promotes tumor growth but also facilitates collagen synthesis and extracellular matrix (ECM) deposition, which stands as one of the principal characteristics of UF pathogenesis [9].

Nevertheless, research on the immunopathogenic mechanisms of UF remains limited, with most studies focusing on local tissue immunity rather than systemic immune responses. While emerging evidence, such as elevated serum IL‐8 levels in UF patients, suggests a role for systemic inflammation in disease progression [10], the functional contribution of peripheral immune cell dysfunction to UF pathogenesis remains poorly defined. Based on these observations, we hypothesize that UF is not merely a localized pathology but a systemic condition involving dysregulated immune responses.

This hypothesis is further supported by the frequent clinical coexistence of UF with endometriosis—another condition characterized by immune dysregulation. In our prior research, which focused on endometriosis, we observed significant alterations in the peripheral immune profiles of affected patients [11]. Notably, we explored the influence of UF on the peripheral immune characteristics of endometriosis patients and observed similarities between the immune profiles of UF and endometriosis, suggesting that fibroids themselves may be linked to systemic immune disturbances. To directly test this hypothesis, we designed the present study to focus specifically on UF without the confounding influence of endometriosis.

Therefore, this study aims to systematically characterize peripheral immune cell profiles in patients with UF but without concurrent endometriosis, compared with healthy controls (HCs). Through this focused investigation, we seek to determine whether UF is associated with a distinct and characteristic peripheral immune imbalance, thereby evaluating its systemic immunological underpinnings and potential as a source of noninvasive biomarkers.

Building on this background, the present study employed flow cytometry to analyze peripheral blood immune function in 31 UF patients and 63 age‐matched healthy female controls. A total of 70 immune cell functional indices were assessed, encompassing T cells, B cells, NK cells, *γ*δ T cells, and their respective functional subsets. By comparing immune characteristic differences between the two groups, we aimed to comprehensively evaluate the peripheral immune function of UF patients. Additionally, we also used the random forest (RF)—machine learning algorithm to construct an immune diagnostic model capable of accurately distinguishing UF patients from HCs. The immune factors incorporated into this model may hold crucial significance in elucidating the immunopathological mechanisms underlying UF. Our research findings would serve as a foundation for in‐depth exploration of its potential involvement in the initiation and progression of the disease.

## 2. Materials and Methods

### 2.1. Study Subjects

A total of 31 patients with UF were recruited in our study from The First People’s Hospital of Foshan (Foshan Hospital Affiliated to Southern Medical University). They underwent various surgical procedures based on individualized clinical decisions, including laparoscopic myomectomy, hysteroscopic myomectomy, and abdominal myomectomy. The choice of procedure was guided by the patient’s age, desire for fertility preservation, symptoms, fibroid characteristics, and surgeon expertise. The inclusion criteria were (1) diagnosed with UF through both ultrasound examination and pathological confirmation, (2) aged between 25 and 45 years (inclusive), (3) with complete medical records, (4) had not received any form of medication in the 3 months prior to surgery, and (5) informed about the study and signed the consent form. The exclusion criteria were (1) having a history of malignancy, chronic inflammatory diseases, infectious diseases, or autoimmune diseases; (2) long‐term use of immunosuppressants and hormonal drugs; (3) serious damage or failure of the heart, liver, kidney, and other important organs; and (4) combined endometriosis and adenomyosis.

HCs were recruited from individuals undergoing routine health examinations at the hospital’s health management center. Inclusion criteria were (1) normal physical examination and laboratory test results within the past year, with pelvic ultrasound confirming the absence of gynecological pathologies, and (2) no self‐reported history of gynecological symptoms or diseases (including UF, adenomyosis, endometriosis, abnormal uterine bleeding, or chronic pelvic pain), as confirmed by standardized clinical interviews and questionnaires. The control group was age‐matched to the patient cohort.

The study protocol was approved by the Ethics Committee for Medical Research of The First People’s Hospital of Foshan (Foshan Hospital Affiliated to Southern University of Science and Technology), and all participants signed written informed consent before enrolling in the study.

### 2.2. Clinical Data Collection

Clinical data were collected for all UF patients, including age, obstetric and gynecological history (e.g., gravidity and parity), clinical symptoms, number of leiomyomas, their location, and the maximum diameter of the largest fibroid.

### 2.3. Peripheral Blood Lymphocyte and Subset Testing

#### 2.3.1. Specimen Collection

Preoperative venous blood samples (3–5 mL) were collected from patients using sodium heparin anticoagulant tubes. All samples were either analyzed immediately after collection or stored at 4 °C and processed within 48 h. The control group samples were collected and handled in the same way.

#### 2.3.2. Flow Cytometry Assay

Each sample tube was prepared with 1 × 10^6^ cells, which were resuspended in phosphate‐buffered saline (PBS). The cells were centrifuged at 1000 rpm for 5 min, and the supernatant was discarded. Next, 10 *μ*L of a fluorescein‐labeled membrane surface antibody cocktail was added and mixed thoroughly (antibodies purchased from BD Biosciences; detailed specifications are provided in Supporting Information 1: Table S1). The mixture was incubated at room temperature for 15 min to allow sufficient binding of antibodies to membrane surface antigens. Subsequently, 1 mL of erythrocyte lysis buffer was added, and the suspension was mixed gently and incubated at room temperature for 10–15 min to achieve complete erythrocyte lysis. After lysis, 2–3 mL of PBS supplemented with 1% bovine serum albumin (BSA) or 2% fetal bovine serum (FBS) (as recommended) was added, followed by centrifugation at 1000 rpm for 5 min; the supernatant was discarded. This washing step was repeated once to remove unbound antibodies. Finally, the cells were resuspended in 350–500 μL of PBS and analyzed on a BD FACSCanto Plus flow cytometer (BD Biosciences, USA; three lasers, ten parameters) within 3 h.

#### 2.3.3. Flow Cytometry Analysis

Data acquisition and analysis were performed using FlowJo software (version 10). The gating strategy began with forward scatter (FSC) and side scatter (SSC) to exclude debris and select lymphocytes. T cells were then identified as CD3^+^ and further subdivided into CD4^+^ helper and CD8^+^ cytotoxic T (Tc) cells. Subsequent phenotyping utilized a panel of surface and intracellular markers—including CD45RA, CCR7, CD28, CD25, CD127, CXCR5, CCR4, CCR6, HLA‐DR, CD38, PD‐1, and CD57—to delineate naïve, central memory, effector memory, terminally differentiated, regulatory (Treg), follicular helper (Tfh), Th1/Th2/Th17, and Tc1/Tc2/Tc17 subsets. *γδ* T cells were gated based on TCR *γδ* expression and further classified using *γδ*1 and *γδ*2 chains along with functional receptors such as NKG2D, NKp30, and NKp46. B cells were initially selected as CD19^+^ and then stratified into naïve, memory, marginal zone (MZ), B10, and plasmablast subsets based on the expression of IgD, IgM, CD27, CD38, CD24, and CD21. NK cells were defined as CD3^−^CD56^+^ and further resolved into immature/mature and functionally distinct populations using CD56^bright/dim^ intensity and markers including NKG2D, NKp30, NKp46, and the CD94/KIR axis. All detailed gating strategies are illustrated in Supporting Information 2: Figures S2–Supporting Information 6: S6. For each immune cell subset, we used percentages within the relevant parent population and absolute counts derived from complete blood counts. These variables were analyzed without additional mathematical transformation. A total of 70 immunological parameters were evaluated in this study.

#### 2.3.4. Quality Assurance in Experiments

The same optical configuration and filter settings were used for all measurements. Instrument performance was verified daily using BD–supplied calibration beads, following the manufacturer’s instructions. Target median fluorescence intensities (MFIs) and coefficients of variation for each channel were monitored, and photomultiplier tube (PMT) voltages were kept constant across all acquisition days once optimal settings had been established. Runs that did not meet the daily quality‐control criteria were repeated after recalibration.

Fluorescence compensation was performed using unstained and single‐stained controls for each fluorochrome. Compensation matrices were generated on the instrument at the time of acquisition and subsequently inspected in FlowJo (BD, USA). The same verified compensation matrix was applied to all samples acquired with the corresponding antibody panel.

Samples were processed in several acquisition batches. Whenever possible, patients with UF and control participants were included in the same batch to reduce confounding by acquisition date. HC samples from the health management center were intermittently included alongside patient samples to monitor major lymphocyte subsets, although a fixed internal reference sample was not available for every batch.

### 2.4. Statistical Methods

Statistical analysis was performed using IBM SPSS 22.0 and GraphPad Prism 8.0 software. Count data were expressed as *n* (%). The normality test was used for measurement data, and the independent samples *t*‐test was used for comparison between groups with normal distribution; the Mann‐Whitney *U* test was used for comparisons between groups with non‐normal distributions. Spearman’s correlation analysis was used to analyze the relationship between immune indicators and age. RF machine learning method and receiver operating characteristics (ROC) analysis were done by R software (version 4.5.1). Two‐sided tests were used for all analyses. To account for multiple comparisons, the false discovery rate (FDR) correction was applied. A corrected *p*‐value (FDR) of <0.05 was considered statistically significant.

## 3. Results

### 3.1. Clinical Characteristics

A total of 31 UF patients and 63 HCs were recruited and enrolled in this study, and all subjects met the inclusion and exclusion criteria. All subjects were women of reproductive age (25–45 years), and there was no significant difference in age between the groups of UF patients and HCs (median [IQR]: 37 [33–42] vs. 36 [32–41] years; Supporting Information 7: Figure S1). The clinical characteristics of patients with UF are shown in Table 1.

**Table 1:** Clinical characteristics of patients with UF.

Total number of cases (*n*)	31
Age (years)	
25–35	9 (29%)
35–45	22 (71%)

Comorbidities (*n*, %)	
Endometrial polyp	7 (22.58%)
Fallopian tube cyst	2 (6.45%)

Number of UF (*n*, %)	
1	15 (48.39%)
2	4 (12.90%)
≥3	12 (38.71%)

Location of UF (*n*, %)	
Submucosal	1 (3.23%)
Intramural	18 (58.06%)
Subserosal	6 (19.35%)
Submucosal + intramural	3 (9.68%)
Intramural + subserosal	1 (3.23%)
Submucosal + intramural + subserosal	2 (6.45%)

Maximum diameter of UF (*n*, %)	
≤5 cm	8 (25.81%)
>5 cm	23 (74.19%)

Clinical symptom (*n*, %)	
Dysmenorrhea	3 (9.68%)
Abnormal bleeding	14 (45.16%)
Other^1^	16 (51.61%)

History of pregnancy (*n*, %)	
Yes	27 (87.10%)
No	4 (12.90%)

^1^Refers to patients without clinical symptoms (e.g., pain, abnormal bleeding, or infertility) that would typically be indicated in the chief complaint, and in whom ultrasound findings suggested a UF.

### 3.2. Analysis Reveals No Significant Differences in CD3^+^ T Cells and Their Subsets Between UF Patients and Controls

**Figure 1:**
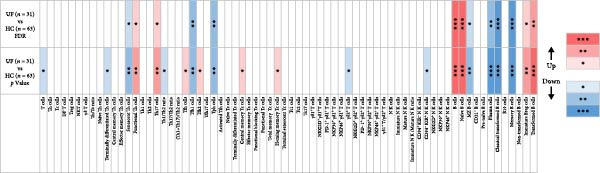
Distribution profiles of peripheral blood immune cell subsets in patients with UF. Red indicates significant upregulation in the UF group compared to the HC group; blue represents significant downregulation. Statistical significance is marked as  ^∗^FDR < 0.05,  ^∗∗^FDR 0.01, and  ^∗∗∗^FDR < 0.001.

We analyzed immune cells from the peripheral blood of 31 UF patients and 63 HCs using flow cytometry assays, containing T cells, B cells, NK cells, *γδ* T cells, and their multiple functional subpopulations, involving a total of 70 different immune cell subpopulations, as shown in Figure 1.

We first conducted a comparative analysis of T cells (CD3^+^) and their subsets in the peripheral blood lymphocytes of UF patients and HCs. The subsets analyzed were helper T cells (Th, CD4^+^), Tc cells (CD8^+^), double‐positive T cells (DP T, CD4^+^CD8^+^), regulatory T cells (Treg, CD4^+^CD25^+^CD127ˡᵒʷ/^−^), NK T cells (NK T, CD56^+^), and *γδ* T cells (*γδ* ^+^). The results showed that the proportion of T cells within lymphocytes and the proportions of these subsets within T cells showed no significant differences (Figure 1 and Supporting Information 8: Table S2).

### 3.3. Dysregulation and Imbalance in Th Functional Subsets of UF Patients

We analyzed and characterized a total of 18 Th functional subsets, including naïve Th (CD45RA^+^CCR7^+^), terminally differentiated Th (CD45RA^+^CCR7^−^), central memory Th (CD4^+^ T_CM_ cells and CD45RA^−^CCR7^+^), effector memory Th (CD4^+^ T_EM_ cells and CD45RA^−^CCR7^−^), senescent Th (CD28^−^), functional Th cells (CD28^+^), as well as Th1 (CXCR5^−^CXCR3^+^CCR4^−^), Th2 (CXCR5^−^CXCR3^−^CCR4^+^), Th17 (CXCR5^−^CXCR3^−^CCR4^−^CCR6^+^), Th1/Th2, Th17/Th2, (Th1+Th17)/Th2, T follicular helper cells (Tfh and CXCR5^+^), Tfh1 (CXCR5^+^CXCR3^+^CCR4^−^), Tfh2 (CXCR5^+^CXCR3^−^CCR4^+^), Tfh17 (CXCR5^+^CXCR3^−^CCR4^−^CCR6^+^), peripheral Th (Tph and CXCR5^−^PD‐1^+^), and activated Tfh cells (CXCR5^+^PD‐1^+^).

The results revealed a significant dysregulation in the functional subsets of Th cells between UF patients and HCs. UF patients exhibited significantly higher proportions of functional Th and Th17 cells while showing significantly lower proportions of senescent Th, Tfh1, and Tph cells (Figure 2 and Supporting Information 8: Table S2).

Figure 2Comparative analysis of Th cell subset expression between UF patients and HC. Statistical significance is denoted as  ^∗^FDR < 0.05,  ^∗∗^FDR < 0.01,  ^∗∗∗^FDR < 0.001. (A) Senescent Th cells. (B) Functional Th cells. (C) Th17 cells. (D) fTh1 cells. (E) Tph cells.

**Figure:**
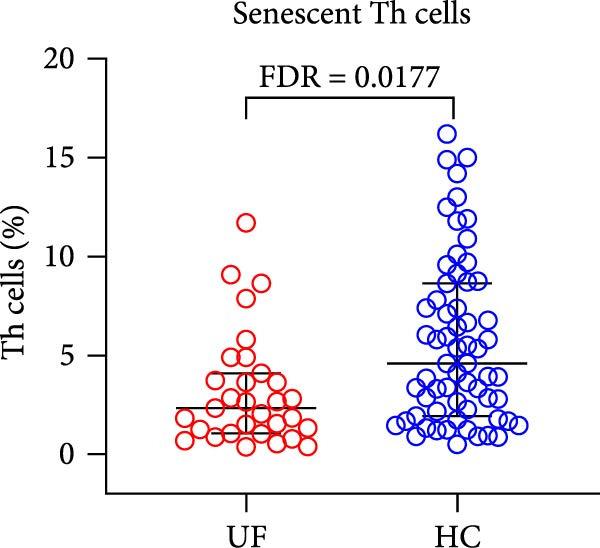
(A)

**Figure:**
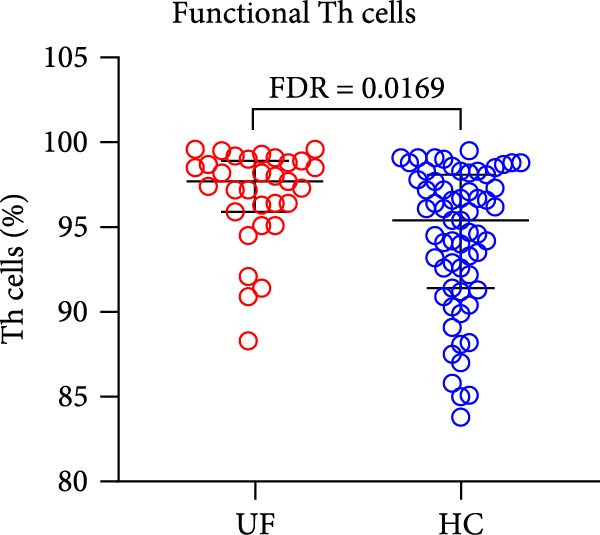
(B)

**Figure:**
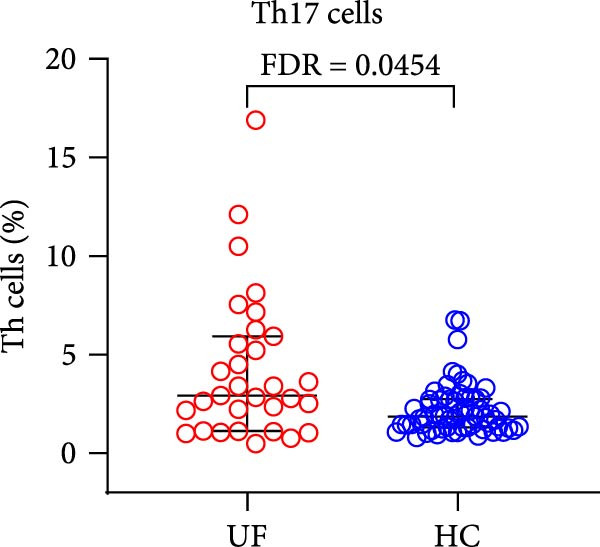
(C)

**Figure:**
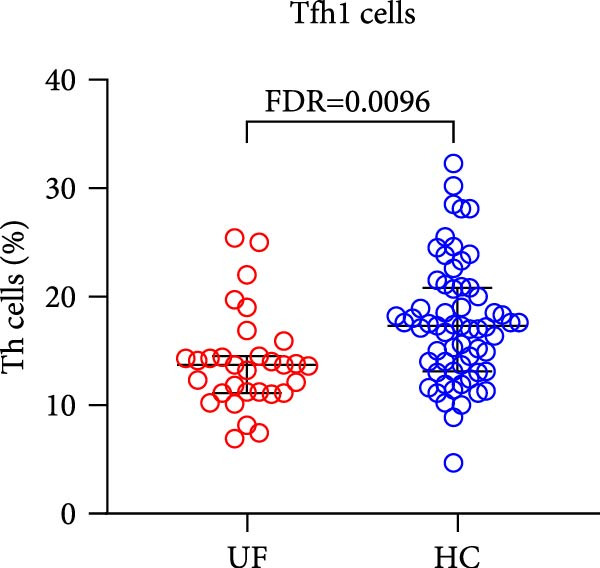
(D)

**Figure:**
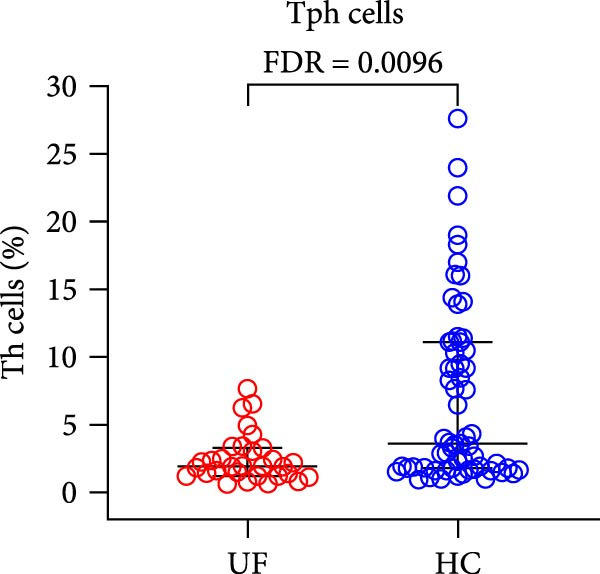
(E)

### 3.4. Peripheral Tc Functional Subsets Are Largely Unaltered in UF Patients

Within the Tc functional subsets, we analyzed and characterized naïve Tc (CD45RA^+^CCR7^+^), terminally differentiated Tc (CD45RA^+^CCR7^−^), central memory Tc (CD8^+^ T_CM_ cells and CD45RA^−^CCR7^+^), effector memory Tc (CD8^+^ T_EM_ cells and CD45RA^−^CCR7^-^), functional blocking Tc (PD‐1^+^), functional Tc (CD28^+^), total memory Tc (HLADR^+^), homing memory Tc (HLADR^+^CD38^+^), terminal senescent Tc cells (CD28^−^CD57^+^), as well as Tc1 (CXCR5^−^CXCR3^+^CCR4^−^), Tc2 (CXCR5^−^CXCR3^−^CCR4^+^), and Tc17 cells (CXCR5^-^CXCR3^−^CCR4^-^CCR6^+^), totaling 12 cell subsets.

Overall, no statistically significant differences were found in any Tc functional subsets between UF patients and HCs (Figure 1 and Supporting Information 8: Table S2). Any observed differences, such as those in central memory and homing memory Tc subsets, were minor.

### 3.5. Peripheral *γδ* T Cells and Their Subsets Are Largely Unaltered in UF Patients

Within the *γδ* T functional subsets, we detected and analyzed the major subsets (*γδ*1^+^ T cells and *γδ*2^+^ T cells) along with their functional subpopulations. These included functionally mature (NKG2D^+^), functionally inhibitory (PD‐1^+^), conventional cytotoxic (NKP30^+^), and virus‐specific cytotoxic populations for both *γδ*1^+^ T cells and *γδ*2^+^ T cells.

Despite minor reduction in the proportions of *γδ*2^+^ T cells, the results indicated no significant differences in the frequencies of *γδ*1^+^ and *γδ*2^+^ T cells or their functional subsets between the groups (Figure 1 and Supporting Information 8: Table S2).

### 3.6. Peripheral NK Cells and Their Subsets Are Largely Unaltered in UF Patients

We also examined the expression levels of NK cells (CD3^−^CD56^+^) and their eight distinct subsets in the peripheral blood of UF patients, including immature NK cells (CD56^bright^), mature NK cells (CD56^dim^), immature/mature NK ratio, early function‐blocking NK (CD94^+^KIR^−^), late function‐blocking NK (CD94^−^KIR^+^), functionally mature NK (NKG2D^+^), conventional cytotoxic NK (NKP30^+^), and virus‐specific cytotoxic NK cells (NKP46^+^).

The results showed that no significant differences were found in the proportion of NK cells or the frequencies of NK subsets between UF patients and HCs, with the exception of a marginally decreased frequency of CD94^−^KIR^+^ NK cells (Figure 1 and Supporting Information 8: Table S2).

### 3.7. Significant Alterations in B Cells and Their Functional Subsets in UF Patients

Finally, we analyzed the expression levels of B cells (CD3^−^CD19^+^) and their 11 functional subsets in the peripheral blood of UF patients, including naïve B cells (CD27^−^IgD^+^), MZ B cells (MZ B cells and CD27^+^IgD^+^), CD21^−^ B cells (CD38^−^CD21^−^), as well as prenaïve B cells (IgD^−^IgM^−^CD27^−^CD38^+^), plasma cells (IgD^−^IgM^−^CD27^+^CD38^+^), classical transformed B cells (IgD^−^IgM^−^CD27^+^CD38^−^), B10 cells (IgD^+^IgM^+^CD27^+^CD24^+^), memory B cells (CD27^+^CD38^−^), nontransformed B cells (IgM^+^CD27^+^CD38^−^), immature regulatory B cells (immature Breg and IgD^+^IgM^+^CD24^+^CD38^+^), and transformed B cells (IgM^+^CD27^−^CD38^+^CD24^+^).

The results showed that, compared with the HC group, the proportion of B cells within lymphocytes was significantly increased in UF patients (Figure 3A). Furthermore, the frequencies of naïve B cells, immature Breg cells, and transformed B cells were significantly elevated (Figure 3B, G, H). In contrast, the percentages of MZ B cells, plasma cells, classical transformed B cells, and memory B cells among total B cells were significantly reduced in UF patients (Figure 3C–F).

Figure 3Comparative analysis of B cell and their subset expression between UF patients and HC. Statistical significance is marked as  ^∗^FDR < 0.05,  ^∗∗^FDR < 0.01,  ^∗∗∗^FDR < 0.001. (A) B cells. (B) Naïve B cells. (C) MZ B cells. (D) Plasma cells. (E) Classical transformed B cells. (F) Memory B cells. (G) Immature Breg cells. (H) Transformed B cells.

**Figure:**
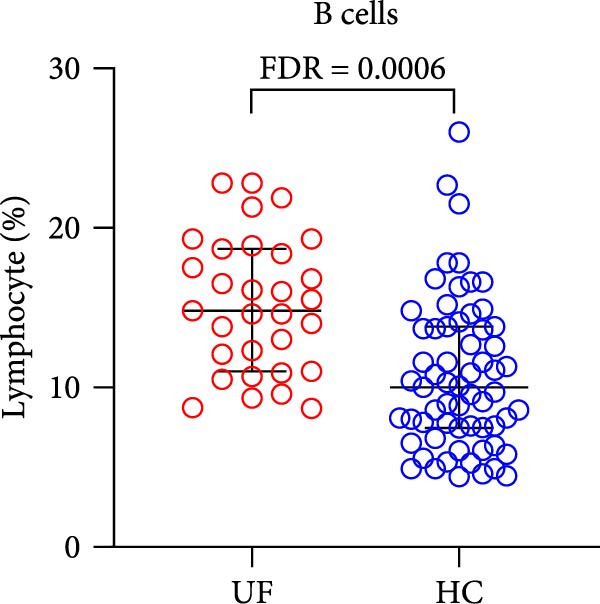
(A)

**Figure:**
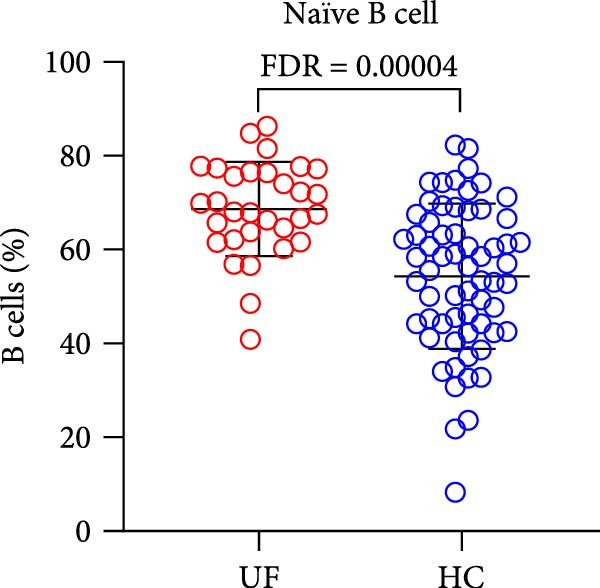
(B)

**Figure:**
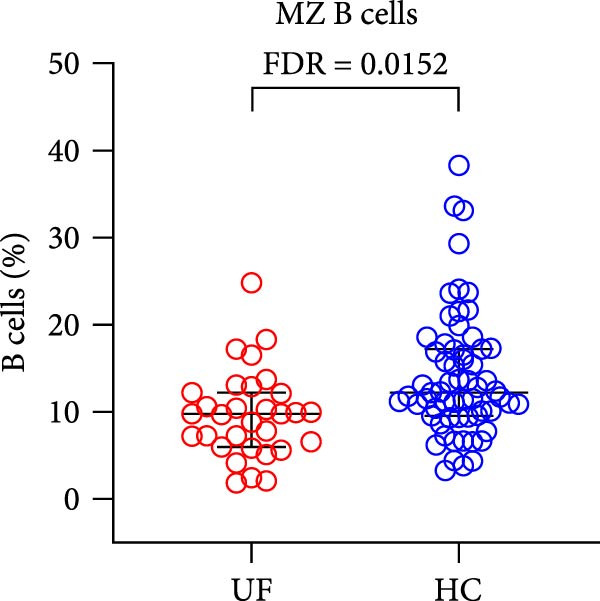
(C)

**Figure:**
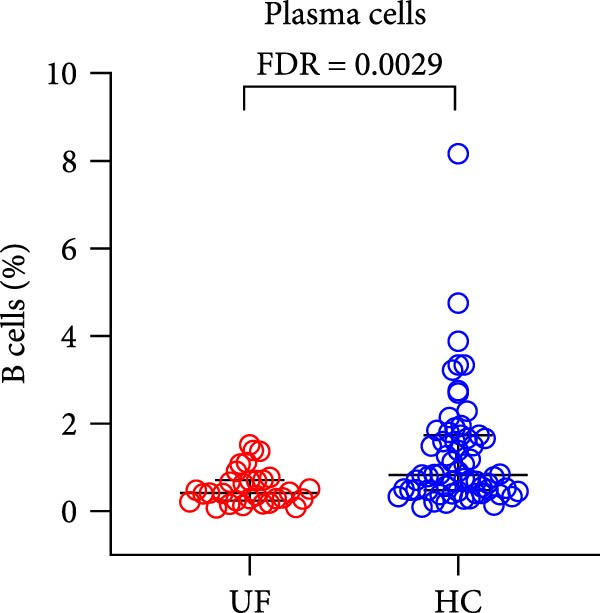
(D)

**Figure:**
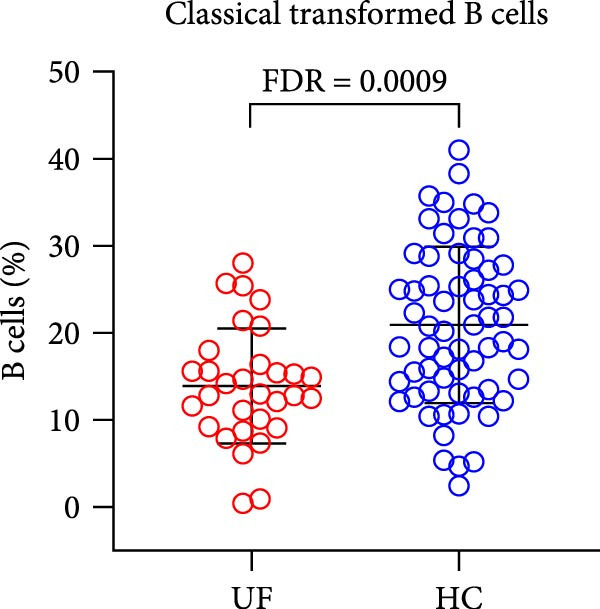
(E)

**Figure:**
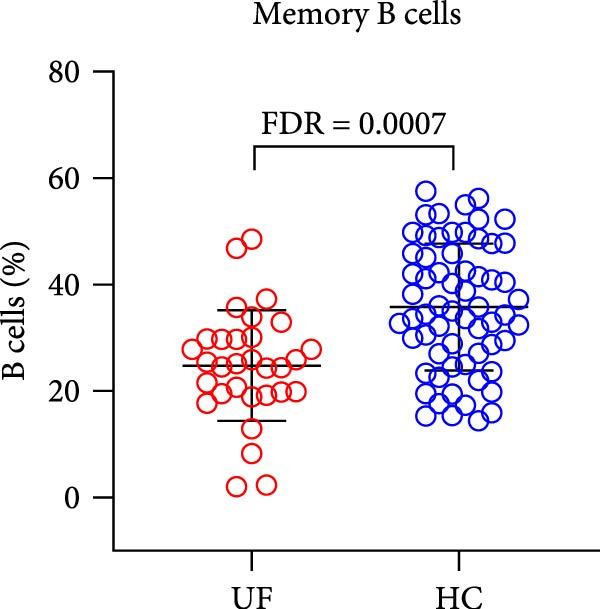
(F)

**Figure:**
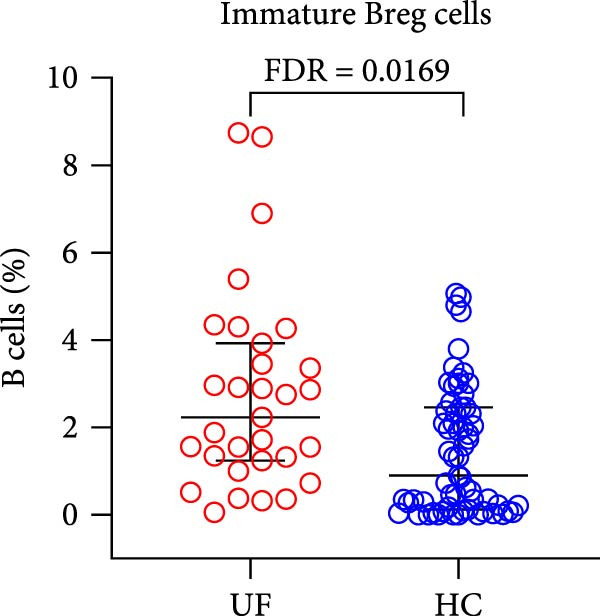
(G)

**Figure:**
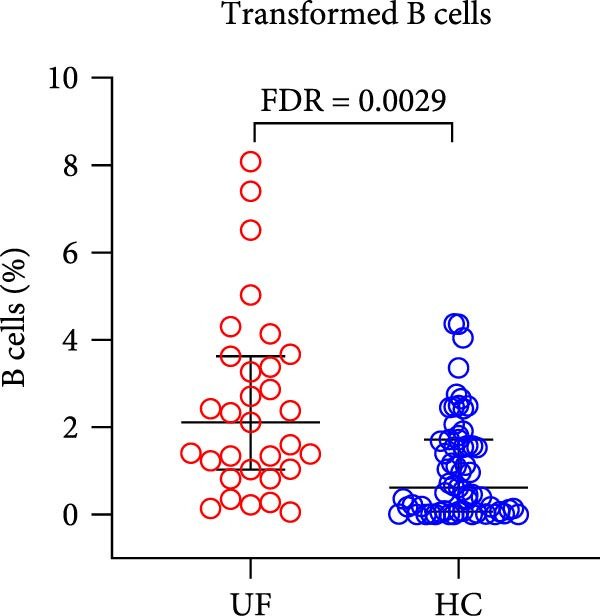
(H)

### 3.8. The Potential Value of Peripheral Immune Markers in the Diagnosis of UF

The above results indicate that the peripheral immunity in patients with UF is significantly different from that of healthy individuals, as demonstrated by notable differences in the 13 peripheral immune markers analyzed. Based on these findings, we further attempted to use the RF machine learning method, incorporating these 13 immune markers, to establish a diagnostic model for distinguishing UF from healthy individuals.

In order to establish a reliable diagnostic model, we first subjected the model to a series of tests involving 500 random samples, which were systematically divided into a training set comprising 75% of the data and a test set encompassing the remaining 25%. This approach allowed us to meticulously evaluate the model’s stability and consistency in making accurate diagnoses across diverse sample subsets, thereby ensuring its robustness and reliability in real‐world applications. The diagnostic ability was evaluated using the area under the curve (AUC) indicator derived from ROC analysis. The model constructed generated an ROC curve with an AUC value of 0.876 (95% CI = 0.711–0.876; Figure 4A). The distribution of AUC values obtained from 500 random samples had an average AUC value of 0.879 (95% CI = 0.714–0.961; Figure 4B). In order to identify key features, the importance ranking of the 13 markers was calculated to screen an RF model (Figure 4C). The results showed that the average Gini coefficient reduction values for markers such as B cells, Tph cells, and naïve B cells were the highest, suggesting that these markers may be core biomarkers driving group differentiation.

Figure 4Development of a diagnostic model for UF using the RF algorithm. (A) ROC curve showing the performance of the RF diagnostic model constructed using 13 peripheral immune markers identified as having significant differences after FDR correction. (B) Distribution of AUC values obtained from 500 random sampling iterations of the model based on the 13 immune markers. (C) Feature importance ranking of the 13 immune markers. (D) Relationship between the number of features used and the corresponding AUC value. (E) Distribution of AUC values from 500 random sampling iterations of the model using the top 6 most important immune markers. (F) Feature importance ranking of the top six immune markers.

**Figure:**
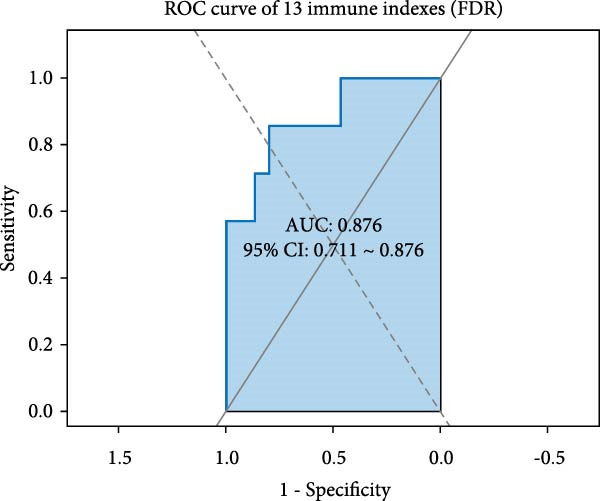
(A)

**Figure:**
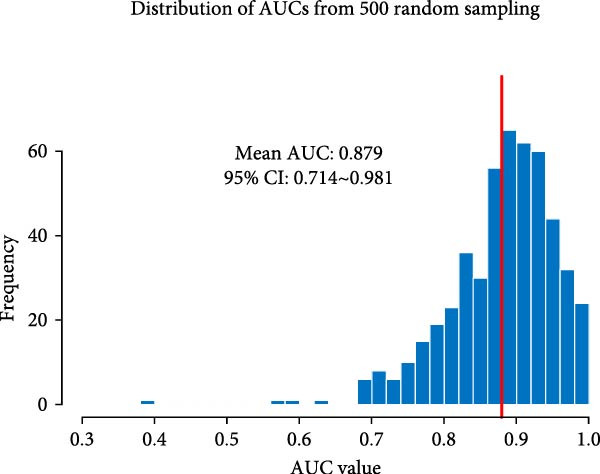
(B)

**Figure:**
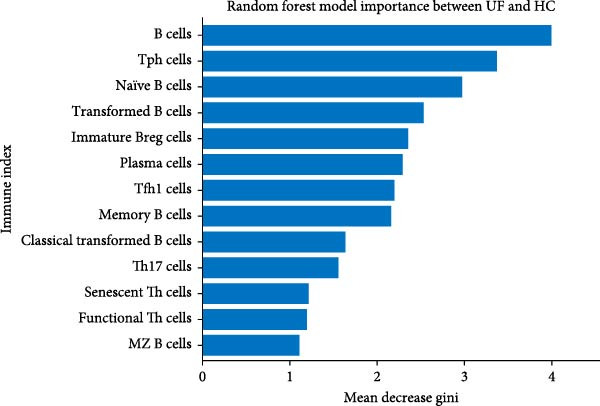
(C)

**Figure:**
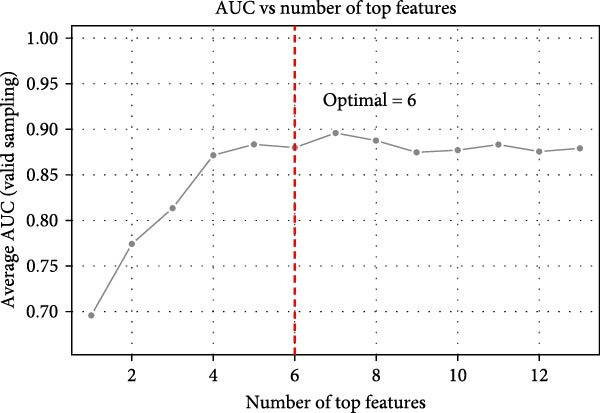
(D)

**Figure:**
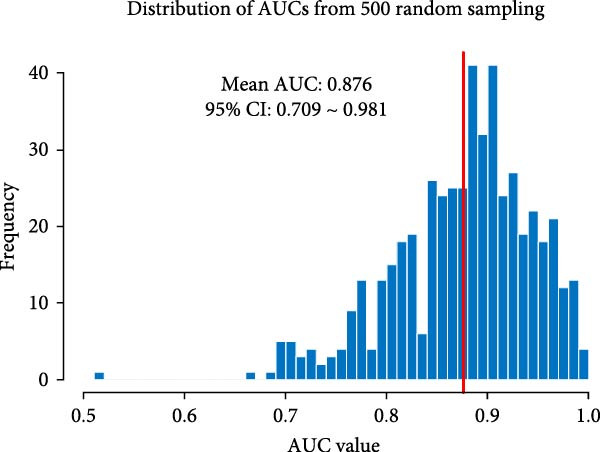
(E)

**Figure:**
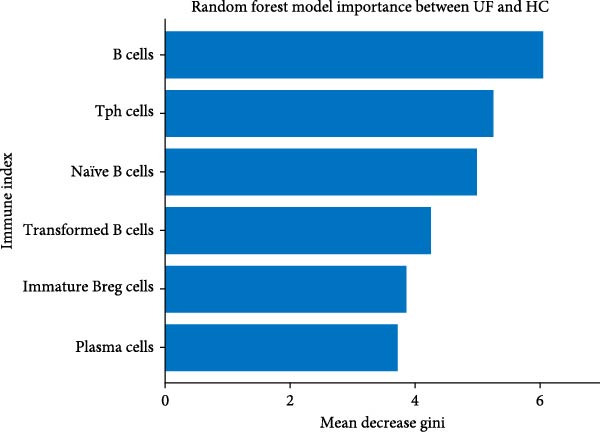
(F)

Based on this, we further explored the optimal feature subset by gradually increasing the number of top features and evaluating their corresponding average AUC. We found that when the top six features were included, the model AUC tended to stabilize (AUC change <0.005; Figure 4D), suggesting that this number is the optimal choice balancing performance and simplicity. The simplified model constructed using these six top features was validated through 500 cross‐validation samples, yielding an average AUC of 0.876 (95% CI = 0.709–0.981, Figure 4E), comparable in performance to the full feature selection model, indicating that the simplified feature subset retains high diagnostic value. Finally, the reordered importance of the top features yielded an identical ranking as in the original model: B cells, Tph, naïve B cells, transformed B cells, immature Breg cells, and plasma cells (Figure 4F).

### 3.9. Relationship Between Peripheral Immune Indicators and Clinical Characteristics in UF Patients

Previous research has demonstrated that the composition and function of peripheral immune cells undergo alterations with advancing age [12]. To begin with, we utilized Spearman’s correlation analysis to determine whether age exerts an influence on peripheral immune markers in both groups. The analysis showed no statistically significant correlation between the 70 indicators and age in either the healthy or the control group, although slight correlations were observed for a few individual indicators within each group (Figure 5A, B). These findings suggest that age is not a major determinant of peripheral blood immune function in our cohorts. This is likely because all participants were women of reproductive age, a demographic with a relatively narrow age range, thereby precluding a substantial age‐dependent effect.

Figure 5Relationship between peripheral immune marker expression and clinical features in patients with UF. (A) Peripheral immune markers showing no significant correlation with chronological age in patients with UF. (B) Peripheral immune markers showing no significant correlation with chronological age in healthy controls. Spearman’s correlation analysis, FDR < 0.05; red, positive correlation; blue, negative correlation. (C) Immune markers are significantly elevated in patients with multiple UF compared to those with a single UF. (D) Immune markers are significantly reduced in patients with multiple UF compared to those with a single UF, FDR < 0.05.

**Figure:**
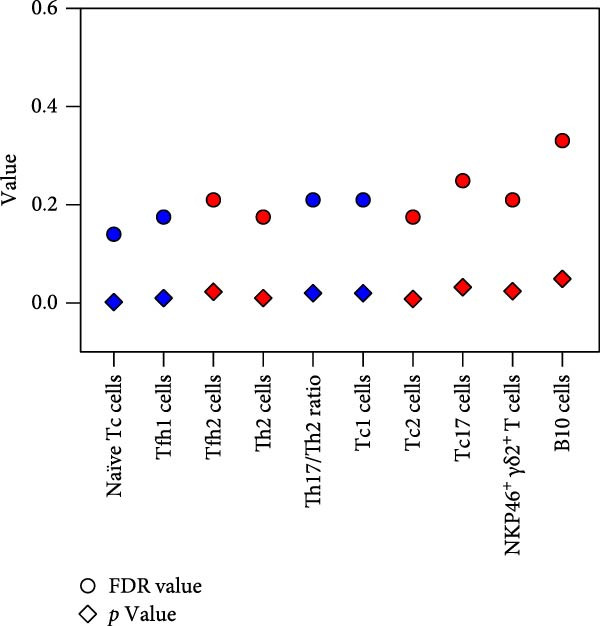
(A)

**Figure:**
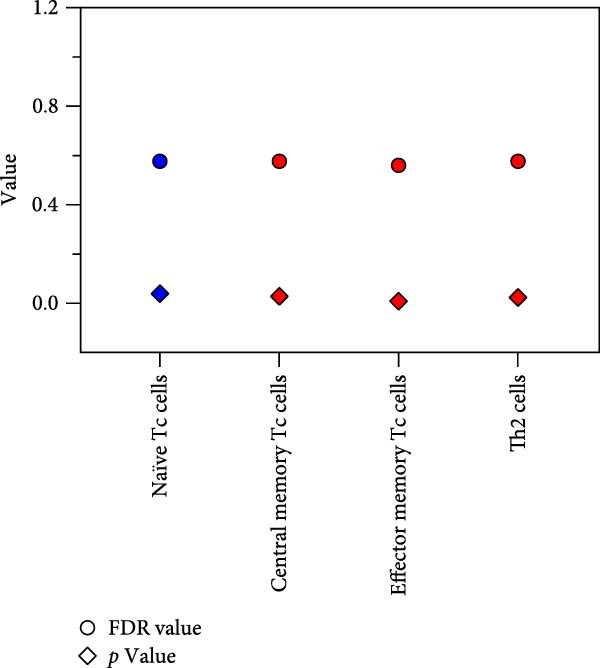
(B)

**Figure:**
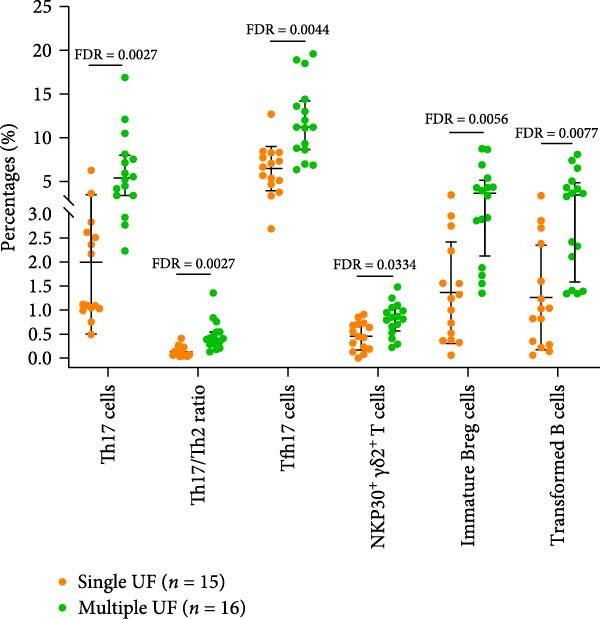
(C)

**Figure:**
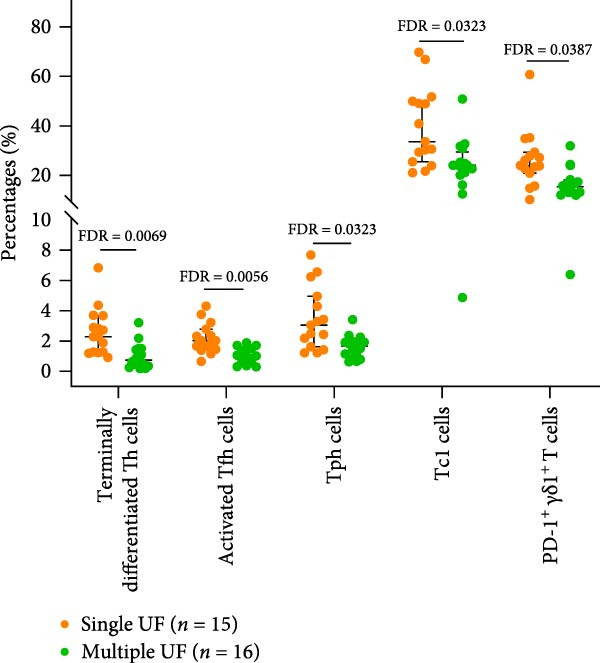
(D)

To investigate associations between peripheral immune markers and clinical features in patients with UF, we compared immune marker expression across groups stratified by fibroid number (single vs. multiple) and the presence of abnormal uterine bleeding. In the comparison between single and multiple UF patients, multiple UF patients had elevated expression levels of Th17, Th17/Th2, Tfh17, NKP30^+^ *γδ*2^+^ T cells, immature Breg cells, and transformed B cells (Figure 5C and Supporting Information 9: Table S3). In contrast, the expression levels of terminally differentiated Th, Tph, activated Tfh, Tc1, and PD‐1^+^ *γδ*1^+^ T cells were significantly decreased in multiple UF patients (Figure 5D and Supporting Information 9: Table S3). However, no statistically significant differences in any indicators were observed between the groups with and without abnormal uterine bleeding symptoms (Supporting Information 10: Table S4).

## 4. Discussion

Traditionally, clinical evaluations of immune function have been confined to examining basic immune cell subsets, including CD3^+^, CD4^+^, and CD8^+^ T cells, monocytes, and neutrophils. However, this conventional methodology falls short in fully uncovering the systemic immune dysregulation associated with diseases. In stark contrast, our study utilized flow cytometry to conduct a systematic analysis of 70 immune cell subsets in the peripheral blood of patients with UF. This approach enabled us to provide a more thorough and accurate portrayal of the peripheral immune landscape in UF patients. These insights, in turn, establish a robust foundation for further exploring the systemic immune dysregulation linked to UF.

In this study, we applied a stringent FDR correction for multiple comparisons to identify differentially expressed immune markers between UF patients and HCs. This procedure confirmed 13 markers with statistically significant differences while excluding nine markers with only marginal differences, as shown in Figure 1, thus providing an initial immunological profile of the peripheral immune landscape in UF.

T cells serve as a pivotal element of the adaptive immune system, primarily orchestrating cellular immune responses and playing a vital role in antitumor immune surveillance. In our current study, we observed only a minimal decrease in T cell expression levels in the peripheral blood of UF patients, with no significant difference compared to controls. Further analysis revealed a compositional shift within the Th cell compartment: Although the total proportion of Th cells remained unchanged, UF patients showed a significant increase in functional Th subsets alongside a marked reduction in senescent Th subsets. Since CD28 is critical for T cell activation and CD28^+^ cells possess enhanced proliferative and multifunctional capacities [13], this immunoprofile reflects a systemic immune system with heightened immunocompetence and more effective surveillance in UF patients. Collectively, these immune alterations may reflect systemic inflammatory processes that promote immune activation and response amplification [5, 14].

T cell differentiation plasticity is crucial for immune regulation. Naïve Th cells can differentiate into functionally distinct effector subsets, such as Th1, Th2, Th17, and Tfh, under the influence of cytokines and other stimuli. These cells participate in different immune responses by secreting specific cytokines [15, 16]. In our cohort, we observed a significant elevation in the frequency of Th17 cells in the peripheral blood of patients with UF. Th17 cells are primarily responsible for secreting cytokines such as IL‐17 and IL‐22. Their core function is to recruit neutrophils and elicit inflammatory responses [17]. The elevated frequency of Th17 cells suggests a sustained, IL‐17–driven proinflammatory state in UF patients [18]. This finding supports the broader immunologic profile of chronic inflammation and functional Th cell activation associated with the disease. Notably, we also observed that Th17 cell levels were significantly more elevated in patients with multiple UF than in those with a single UF, suggesting that the former group may experience a more pronounced systemic inflammatory state.

Tfh cells, a specialized Th subset, are central to germinal center formation and humoral immune response development, establishing durable humoral immunity by regulating B cell proliferation, differentiation, and antibody class switching [19, 20]. Tfh cells can be divided into Tfh1, Tfh2, and Tfh17 subpopulations based on secreted cytokines. Tfh1 cells produce IFN‐*γ* to promote IgG2a production and enhance cellular immune responses [20, 21], while Tfh2 cells secrete IL‐4 to promote IgG1 and IgE production and strengthen humoral immunity [22]. Our study revealed a significant decrease in Tfh1 levels in UF patients. In contrast, total Tfh and Tfh2 levels showed only a mild, nonsignificant increase. This specific reduction in Tfh1 may compromise the immune system’s ability to recognize and restrain fibroid cell proliferation. Collectively, these findings indicate a dysregulation of the adaptive immune response in UF, characterized by a shift away from Type 1 (antiviral/cellular) immunity toward a Type 2 (humoral/profibrotic) bias. This Tfh imbalance reflects an underlying Th1/Th2 disparity in the body, thereby placing UF within the established framework of Type 2 immunity‐driven fibrotic diseases, such as idiopathic pulmonary fibrosis, systemic sclerosis, and certain liver fibroses [23–25].

Another significant finding was the reduced Tph levels in UF patients. Tph activates B cells by secreting IL‐21 and CXCL13 and is positively correlated with inflammatory and malignant disease progression [26, 27]. The reduction in Tph levels signifies a peripheral immune regulatory imbalance in UF patients, manifesting as a comparatively subdued inflammatory response. This compromised inflammatory reaction, which is pivotal for defense and tumor suppression, may thus indicate a failure in immunosurveillance, thereby potentially allowing for the pathological persistence of fibroid cells. Furthermore, we observed a more pronounced decrease in Tph cells in patients with multiple fibroids compared to those with a single fibroid, suggesting a greater degree of inflammatory suppression in the former group.

B cells are vital effector cells in the adaptive immune system, regulating innate immunity via antibody production, antigen presentation, and cytokine secretion. This study reveals significant abnormalities in the proportions of peripheral blood B cells and their subsets in UF patients, a finding that echoes the concurrent dysregulation in Tfh and Tph cells, which are critical for mediating B cell responses. Our study observed elevated levels of naïve B cells, immature Breg, and transformed B cell subsets in the peripheral blood of patients with UF. Naïve B cells, as antigen‐naïve cells, may indicate persistent antigen stimulation or B cell developmental issues [28, 29]. Immature Breg cells secrete immunosuppressive factors like IL‐10, potentially fostering an immune‐tolerant environment [30]. Transformed B cells, at a specific maturation stage, regulate antibody diversity through class switching. These changes collectively suggest widespread dysregulation in B cell development and activation in UF patients. Conversely, UF patients show significant reductions in MZ B cells, plasma cells, classical transformed B cells, and memory B cells. MZ B cells rapidly clear bloodborne antigens [31, 32]; plasma cells, as terminally differentiated effectors, secrete high‐affinity antibodies for pathogen clearance [33]; memory B cells provide long‐term immune protection [34]. Their reduction implies B cell terminal differentiation disorders, impairing antibody‐mediated immune defense in UF patients. Notably, the levels of immature Bregs and transformed B cells were even more elevated in patients with multiple fibroids, indicating a positive correlation between the degree of immune abnormality and the severity of the disease, as measured by the number of fibroids.

Following FDR correction, none of the differences in Tc, *γδ* T, NK cells, or their subsets remained statistically significant, with only minimal differences detected in a few subsets, such as central memory Tc cells, homing memory Tc cells, *γδ*2^+^T cells, and CD94^−^KIR^+^ NK cells. Whether functional dysregulation of these immune cells occurs in the peripheral blood of UF patients requires systematic exploration and validation in future studies.

Additionally, through the application of machine learning techniques, we have pinpointed crucial immune markers essential for developing diagnostic models capable of accurately differentiating UF patients from healthy individuals. These markers include B cells, Tph, naïve B cells, transformed B cells, immature Breg cells, and plasma cells. All the key factors collectively point consistently to peripheral B cell dysregulation as a prominent and pivotal feature of UF, highlighting its translational promise as a key target for novel therapeutic and diagnostic strategies.

This study represents the first attempt to employ flow cytometry for the detection of 70 immune markers in the peripheral blood of UF patients, thereby comprehensively delineating the circulating immune landscape of this patient cohort. This endeavor holds the potential to offer fresh perspectives on the immunological underpinnings of UF pathogenesis. It is important to transparently contextualize our findings within the framework of our related research [11], which utilized the same experimental platform to profile peripheral immune dysregulation in endometriosis. While our prior work identified immunological parallels between patients with endometriosis and those with UF, implying a possible common immune basis, its primary objective remained the characterization of endometriosis itself, which UF served as a comorbid condition of interest. The present study was specifically designed to test the hypothesis derived from that observation: that UF alone, independent of endometriosis, is associated with a distinct peripheral immune signature. By rigorously analyzing a cohort comprising only UF patients without concurrent endometriosis alongside matched HCs, our results not only confirm but also extend the earlier findings. This focused approach allows us to delineate a UF–specific immune profile, thereby providing clearer insight into the systemic immunology of UF, free from the confounding influence of endometriosis.

Nevertheless, this study has certain limitations. First, although rigorous participant screening is essential, our HCs did not undergo MRI or surgical confirmation to rule out underlying gynecological pathologies such as endometriosis or asymptomatic fibroids. This limitation might have potentially introduced a bias in the group comparisons and influenced the results.

Second, this study was confined to the peripheral immunophenotype of UF and did not extend to the local tumor microenvironment. Consequently, we cannot establish a direct relationship between the systemic immune profile and the intratumoral immune landscape, which is crucial for a mechanistic understanding of the disease. Previous studies of the fibroid tissue microenvironment demonstrate a consistent pattern: an elevated presence of innate immune cells (NK cells, macrophages, and dendritic cells) concurrently with a reduced population of adaptive immune cells (notably CD3^+^ and CD4^+^ T cells), relative to matched myometrium [7]. However, in contrast to the enriched NK cell population reported within the fibroid tissue, our peripheral blood analysis did not reveal a significant increase in NK cell levels. These findings provide preliminary evidence that the immune alterations we observed in circulation are only partially mirrored by those reported within the fibroid tissue, a relationship that must be validated in future studies.

Third, the cohort size in this study was ultimately limited, a consequence of our stringent exclusion criteria designed to minimize confounding effects from coexisting conditions such as endometriosis or adenomyosis. While necessary for cohort purity, this smaller sample size may affect the robustness of our findings. Future studies with larger cohorts will be essential to validate these results and enhance the generalizability of the conclusions. Consequently, the predictive model we developed, along with the feature importance rankings derived from it, should be regarded as preliminary. These results are best interpreted as a source of biological hypotheses for future validation, rather than as definitive conclusions. Furthermore, expanding the sample size to include patients from different clinical subgroups—stratified by factors such as fibroid size, location, and the presence of symptoms like dysmenorrhea—would be of significant clinical value. Such investigations could reveal whether peripheral immune profiles vary across these subgroups, thereby offering deeper insights into the heterogeneity of the disease.

Finally, the data gathered thus far are insufficient to fully unravel the immunopathogenic mechanisms of UF. Further research is warranted to delve into the mechanisms through which immune dysregulation and immune interactions precipitate the onset and progression of UF. Such concerted efforts may ultimately pave the way for enhanced clinical diagnosis and treatment modalities for this condition.

## Ethics Statement

This study was approved by the Ethics Committee of the First People’s Hospital of Foshan City (Approval No. [2023]182).

## Consent

All participants provided written informed consent.

## Conflicts of Interest

The authors declare no conflicts of interest.

## Author Contributions

Pei‐Xian Li and Kai‐Rong Lin contributed to study design, data analysis, manuscript drafting, and revision. Si‐fei Yu conducted the experimental work. Yu‐Bin Han and Xiao‐hong Zhu participated in data acquisition, analysis, and interpretation. Pei‐Xian Li and Si‐fei Yu contributed equally to the work.

## Funding

The study was supported by the Guangdong Basic and Applied Basic Research Foundation Enterprise Joint Fund (2021A1515220155) and the Guangdong Medical Research Fund (B2022123).

## Supporting Information

Additional supporting information can be found online in the Supporting Information section.

## Supporting information


**Supporting Information 1** Table S1: Information related to monoclonal antibodies.

**Supporting Information 2** Figure S2: The representative dot diagrams and histograms showed the gating strategy of T cells, Th cells, and Tc cells, as well as the gating strategy of the functional subsets of Th and Tc cells.

**Supporting Information 3** Figure S3: The representative dot diagrams showed the gating strategy of Th and Tc cell functional subsets.

**Supporting Information 4** Figure S4: The representative dot diagrams and histograms showed the gating strategy of *γδ* T cells and their functional subsets.

**Supporting Information 5** Figure S5: The representative dot diagrams and histograms showed the gating strategy of NK cells and their functional subsets.

**Supporting Information 6** Figure S6: The representative dot diagrams showed the gating strategy of B cells and their functional subsets.

**Supporting Information 7** Figure S1: Age distribution comparison between the UF group and the healthy control (HC) group.

**Supporting Information 8** Table S2: Comparison of 70 immune indicators between UF and HC.

**Supporting Information 9** Table S3: Comparison of 70 Immune Indicators between patients with single and multiple UF.

**Supporting Information 10** Table S4: Comparison of 70 Immune Indicators between UF patients with and without abnormal bleeding.

## Data Availability

The supporting data of this study are available from the corresponding author on reasonable request.
