# Supplementary figures and images for: Systematic Analysis of Peripheral Immune Signatures and Diagnostic Model Construction in Patients With Uterine Fibroids
Source: J Immunol Res. 2026 Jan 17;2026:9688793. doi: 10.1155/jimr/9688793 (PMC13140914; doi:10.1155/jimr/9688793)

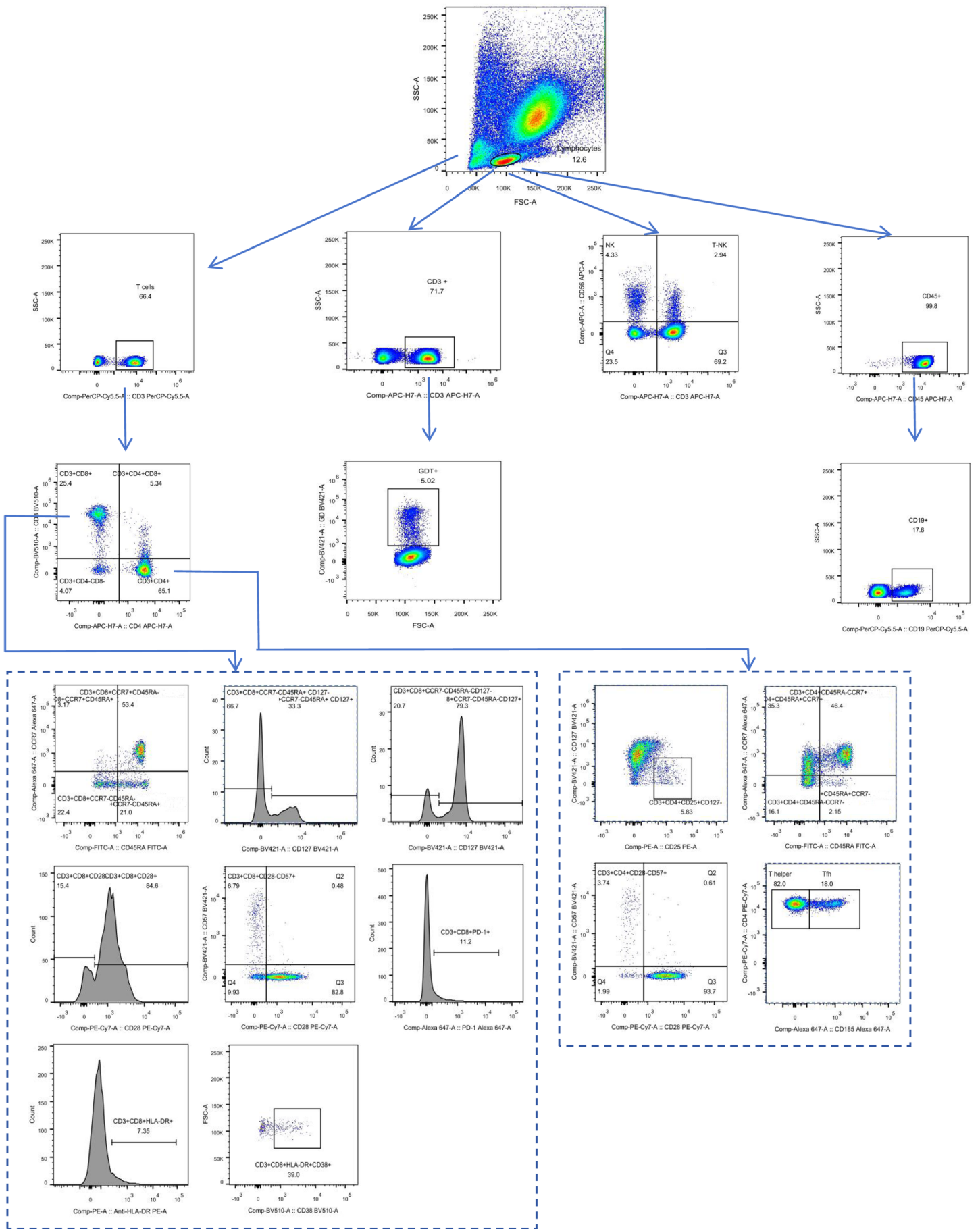

Supplement: Supplementary file 2 — Supporting Information 2 Figure S2: The representative dot diagrams and histograms showed the gating strategy of T cells, Th cells, and Tc cells, as well as the gating strategy of the functional subsets of Th and Tc cells. [file JIMR-2026-9688793-s009.pdf]

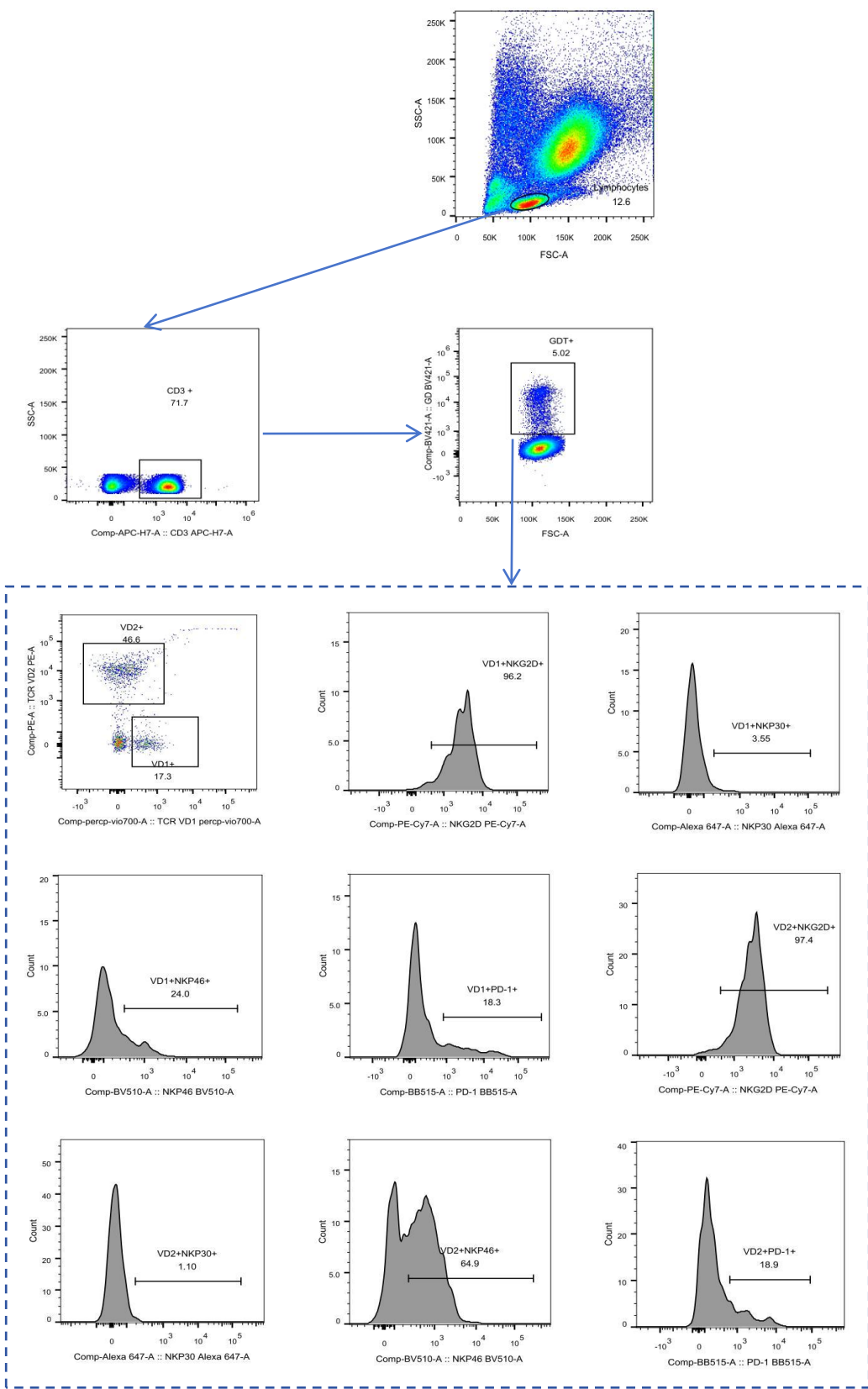

Supplement: Supplementary file 3 — Supporting Information 3 Figure S3: The representative dot diagrams showed the gating strategy of Th and Tc cell functional subsets. [file JIMR-2026-9688793-s002.pdf]

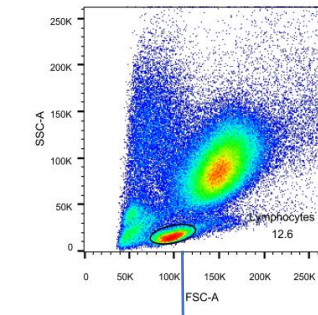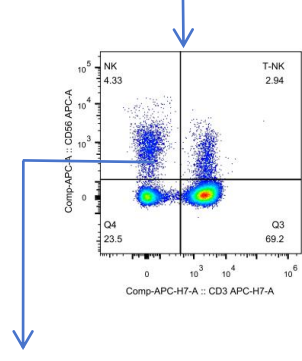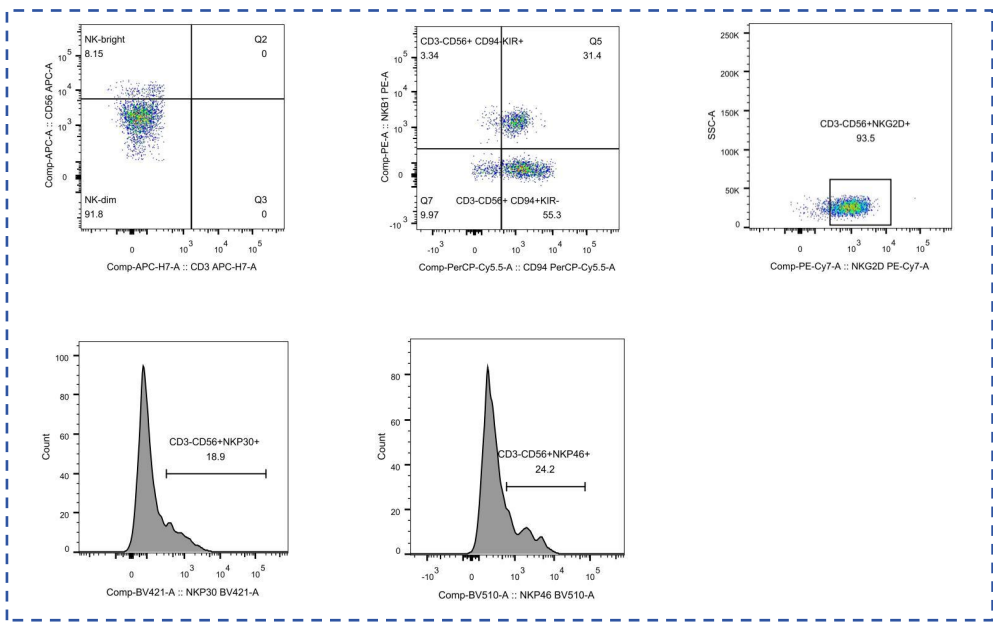

Supplement: Supplementary file 4 — Supporting Information 4 Figure S4: The representative dot diagrams and histograms showed the gating strategy of γδ T cells and their functional subsets. [file JIMR-2026-9688793-s003.pdf]

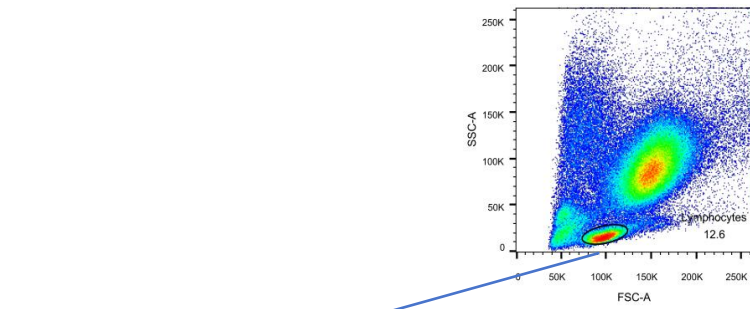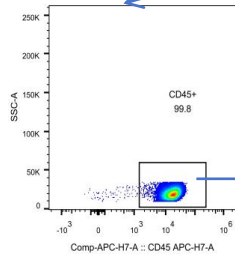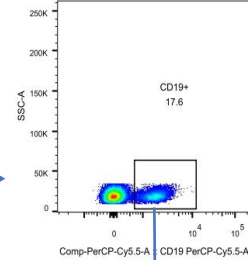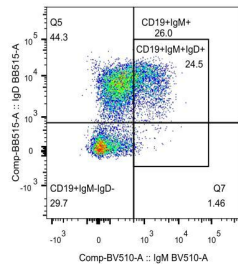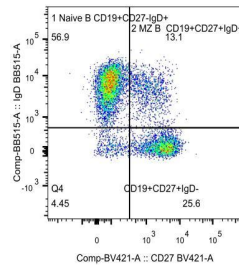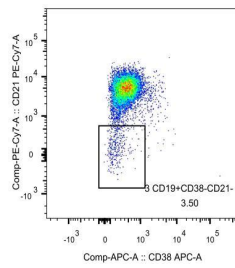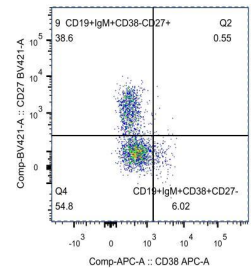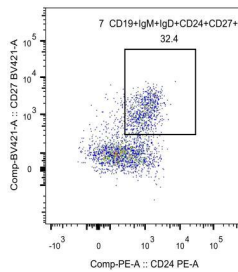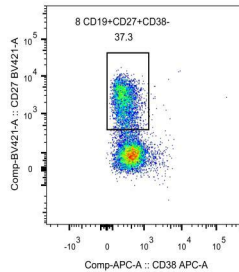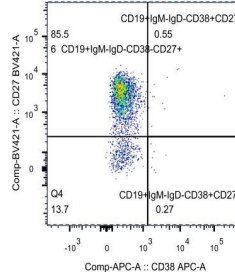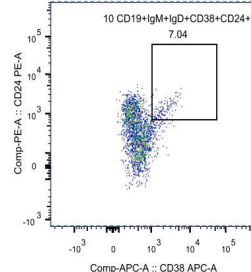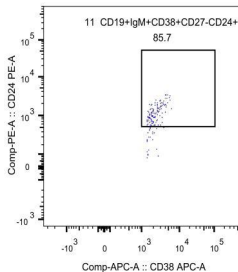

Supplement: Supplementary file 5 — Supporting Information 5 Figure S5: The representative dot diagrams and histograms showed the gating strategy of NK cells and their functional subsets. [file JIMR-2026-9688793-s010.pdf]

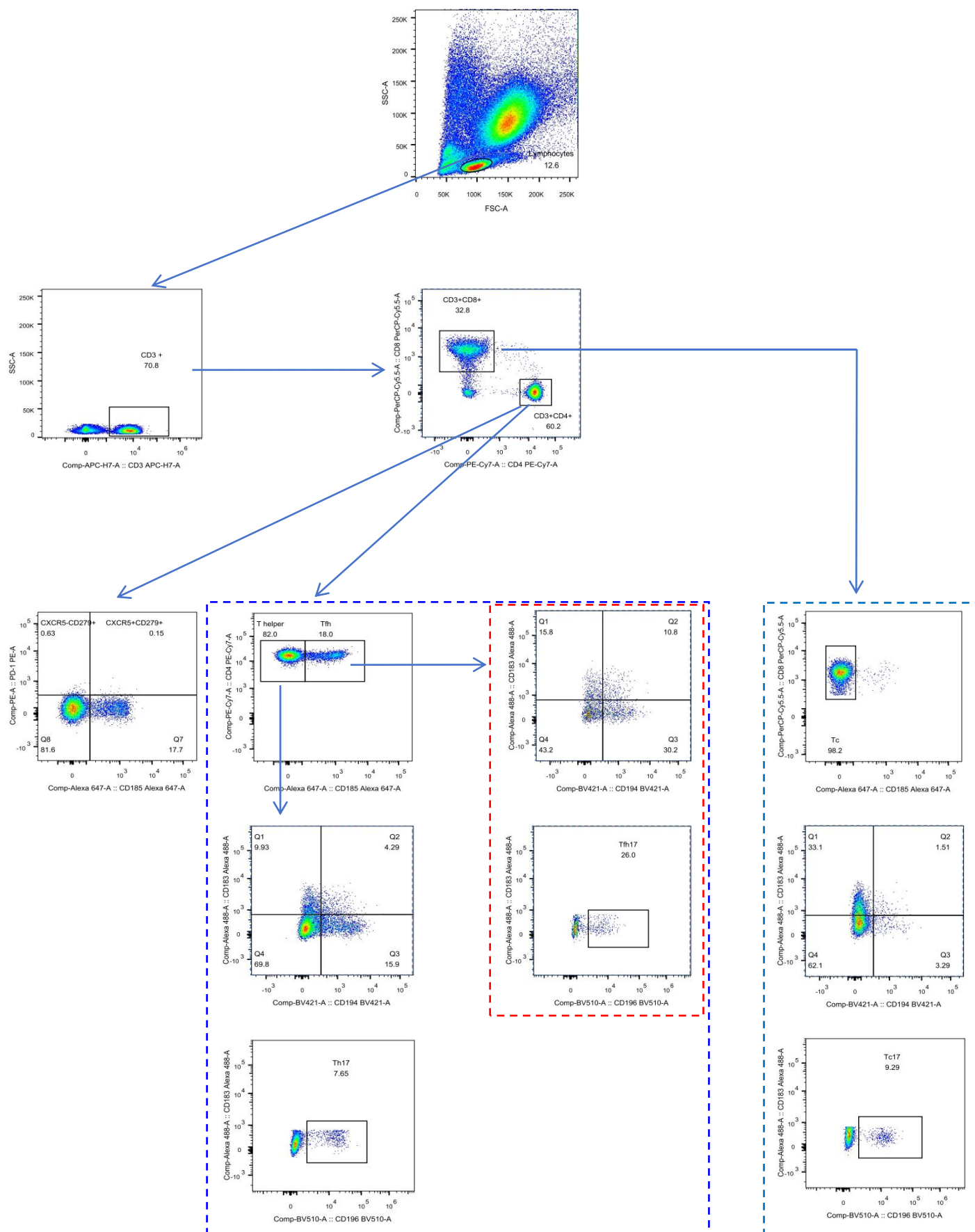

Supplement: Supplementary file 6 — Supporting Information 6 Figure S6: The representative dot diagrams showed the gating strategy of B cells and their functional subsets. [file JIMR-2026-9688793-s005.pdf]

# Age

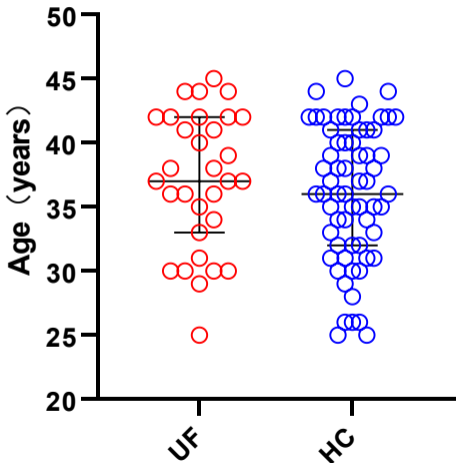

Supplement: Supplementary file 7 — Supporting Information 7 Figure S1: Age distribution comparison between the UF group and the healthy control (HC) group. [file JIMR-2026-9688793-s006.pdf]
